# The abdominal-transhiatal surgical approach versus the thoracoabdominal surgical approach in Siewert type II adenocarcinoma of the esophagogastric junction: protocol for a multicenter prospective, open, parallel, and randomized controlled trial

**DOI:** 10.1186/s12885-022-09375-w

**Published:** 2022-03-24

**Authors:** Qiying Song, Xiongguang Li, Di Wu, Shuo Li, Tianyu Xie, Yixun Lu, Liyu Zhang, Ziyao Xu, Lu Liu, Xin Guo, Xinxin Wang

**Affiliations:** 1 Medical School of Chinese PLA: Chinese PLA General Hospital, Beijing, 100853 China; 2 School of Medicine, Nankai University, Tianjin, 300071 China; 3 Air Force Medical University Xijing Hospital: Xijing Hospital, Xi’an, 710000 China; 4 Department of General Surgery, Chinese PLA General Hospital, Beijing, 100853 China

**Keywords:** Adenocarcinomas of the esophagogastric junction, Siewert type II, Abdominal-transhiatal, Thoracoabdominal

## Abstract

**Background:**

To date, Siewert type II adenocarcinoma of the esophagogastric junction (ST-II AEG) can be removed radically utilizing either the abdominal-transhiatal (TH) or the right thoracoabdominal (RTA) approaches. Because of a paucity of high-quality direct evidence, the appropriate surgical approach for ST-II AEG remains debatable. In the present, only several retrospective studies are available, representing ambiguous results. Thus, prospective randomized clinical trials are demanded to compare the survival, oncological outcomes, safety and efficiency and life quality between the TH and RTA approach in patients with resectable AEG of Siewert type II.

**Methods:**

A prospective, multicenter, open, randomized, and parallel controlled study named S2AEG will be conducted. Three hundred and twelve patients who match the inclusion criteria but not the exclusion criteria will be participating in the trial and randomly divided into the TH (156) and RTA (156) cohorts. The primary efficacy endpoint is the 3-year disease-free survival (DFS) following the operation. The rate of R0-resection, the number and site of lymph nodes infiltrated and dissected, postoperative complications, hospital days and life quality are the second endpoints.

**Discussion:**

This study is the first prospectively randomized controlled trial aiming to compare the surgical outcomes between TH and RTA approaches in patients with resectable ST-II AEG. It is hypothesized that patients in the TH cohort would harvest equivalent oncological results and survival while maintaining acceptable life quality when compared to patients in the RTA cohort. Our findings will provide high-level clinical evidence for clinical decision-making on the appropriate surgical approach for patients with ST-II AEG. Embarked in November 2019, this research will be completed 3 years after the final participant’s enrolment date.

**Trial registration:**

Clinical Trial.gov ID: NCT04910789 May 29, 2021. Name: S2AEG.

## Background

Adenocarcinoma of the esophagogastric junction (AEG) was defined as the tumor whose epicenter was located 5 cm above and below the junction of the esophagogastric junction (EGJ) [1]. In the duration of the past thirty years, the incidence of AEG has a tremendous increase in both Eastern and Western countries [2–6]. Take America for example, there has been an approximately 2.5-fold elevation in the past 35 years [6]. There is a high incidence of gastric cancer in Asian countries, and a single-center large-capacity study carried out by the West China Medical Center also showed that the proportion of AEG in China increased from 22.3% to 35.7% from 1988 to 2012 [7]. Because of the particularities of the anatomical location and biological behavior, Siewert type II AEG (ST-II AEG) with an epicenter located in 1 cm above to 2 cm below the EGJ, is characterized by a higher degree of differentiation, deeper invasion, higher metastasis rate and worse prognosis compared with other sites of gastric cancer [8]. After the operations with further therapy, the patients’ 5-year overall survival rate remains weak and staggers at around 37.9% to 52.3% [9–13]. Curative surgical intervention is still recognized as the cornerstone of multimodal treatment strategies. Therefore, determining the optimal treatment is imperative to improve the patients’ prognoses.

In order to provide the best therapeutic schedule for ST-II AEG patients, a suitable surgical strategy should ensure both complete resection of the original tumor and adequate dissection of the regional lymph node. Regarding the prevalently adopted Siewert classification worldwide, most researchers have reached a consensus that for patients with ST-I AEG and ST-III AEG the optimal surgical approaches are the transthoracic and abdominal-transhiatal (TH) approaches, respectively [1, 14–16]. However, a constantly controversial opinion is illustrated on the optimal surgical approach taken by ST-II AEG [1, 17, 18]. Some experts prefer the TH approach [10, 12], while others favor the thoracoabdominal (TA) approach [19, 20]. Additionally, the surgical methods of the TA approach differ in Eastern and Western countries. Western countries where the standard approach is thoracotomy on the right side (Ivor–Lewis) (RTA), while Asian countries traditionally are apt to the approach of thoracotomy on the left side (LTA). Previous studies have shown that the TA approach can obtain sufficient safety proximal margins and adequate mediastinal lymph node dissection, while harboring the higher postoperative complication rate as well as longer hospital stay [10, 12, 19, 21, 22]. The more vital part is investigating the effects on prognosis. For ST-II AEG, the optimal surgical approach remains unclear considering the oncological outcomes, quality of life (QOL) and survival, as few high-quality randomized controlled trials have been conducted presently [12, 23, 24]. Moreover, most of the available studies are heterogeneous designs and investigate different surgical approaches including not only ST-II but also ST- I or III [12, 23, 24]. No randomized controlled trials (RCTs) on the RTA and TH approaches have been published so far except for an RCT comparing the TH and LTA approaches [10]. It remains a hot point in dispute in present guidelines without clear recommendation [25].

Based on the above, a prospective, multicenter, randomized, and parallel controlled trial named S2AEG is designed to assess the survival, oncology outcomes, and QOL between the TH and RTA surgical approaches for resectable ST-II AEG.

## Methods

### Objective

This clinical trial is oriented toward comparing the safety, feasibility, and clinical efficacy of the TH and RTA surgical approaches for resectable ST-II AEG. The primary endpoint is defined as the 3-year disease-free survival (DFS), and the secondary endpoints include the rate of R0-resection, the number and station of lymph nodes (LNs) dissected and infiltrated, postoperative complications, duration of hospitalization and QOL. It is hypothesized that the TH approach would produce undifferentiated oncological results and survival for patients with ST-II AEG when compared to those with the RTA approach, while still harvesting satisfactory QOL.

### Study design

S2AEG is designed as a prospective, multicenter, randomized, open, and parallel controlled study conducted in China. This trial is funded by the Program for the Bethune Charitable Foundation (2019–12) and has been registered on Clinical-Trial.gov (NCT04910789, named S2AEG). All the Institutional Ethics Review Committees of participating research centers have approved this protocol. Any amendments that may affect the trial will be further communicated to obtain approval among the Institutional Ethics Review Committees. All researchers will conduct this trial in line with the Declaration of Helsinki and Good Clinical Practice guidelines. Before recruitment, each participant will provide written informed consent for the acquisition and use of anonymized clinical data. This study will be conducted by surgeons who are competent in performing both approaches. None of the patients, surgeons, or data analysts will be blinded.

### Study population

The clinical efficacy of the TH and RTA approaches for the resectable ST-II AGE, which is the aim of the study. The eligibility of all patients will be determined by their surgeon.

The inclusion criteria are described briefly below:

1. Aged 18–75;
2. Histologically confirmed as Siewert type II AEG;
3. Clinical stage ranges cT1-4aN0-3 M0 (According to the 8th AJCC TNM staging system) and is confirmed by the preoperative examinations (Gastroscopy, enhanced or not computer tomography (CT), magnetic resonance imaging (MRI) or positron emission tomography with CT (PET-CT)).
4. The operation will be performed by either TH or RTA approach according to the direction of the research center and the surgeon;
5. American Society of Anesthesiologists (ASA) physical status score of 1–3;
6. Eastern Cooperative Oncology Group (ECOG) performance status of 0–1;
7. Laboratory test criteria: hemoglobin > 90 g/L, white blood cell > 3 × 10^9/L, platelets > 100 × 10^^9^/L, serum albumin (ALB) > 30 g/L, renal function (serum creatinine (Scr) < 1.5 × ULN and glomerular filtration rate > 60 ml/min), and liver function (alanine transaminase (ALT) and aspartate transaminase (AST) less than 2.5× upper limit of normal);
8. Informed consent of patients is required.

The exclusion criteria are as follows:

1. Siewert type I or III AEG;
2. Postoperative pathological types confirmed as non-adenocarcinoma or non-simple adenocarcinoma;
3. Distant metastasis (M1) or invasion of the peritoneum;
4. Clinically significant central nervous system disease that may influence compliance;
5. Clinically significant diseases (Irregularly controlled diabetes and hypertension; History of myocardial infarction or cerebral infarction within 6 months before the operation; Heart function: New York Heart Association (NYHA) class II or higher; Respiratory function: Forced expiratory volume in the first second (FEV1) < 50%; Liver function: Child-Pugh class C; Renal function: Serum creatinine (Scr) > 1.5xULN and glomerular filtration rate < 60 ml/min;
6. History of gastrectomy (including endoscopic mucosal resection or submucosal dissection);
7. Diagnosed with other malignancies within 5 years;
8. Pregnancy or breast-feeding;
9. Tumor-related complications (dysphagia, gastrointestinal bleeding, perforation, obstruction, etc.) requiring emergency surgery;
10. Being assessed by the investigator as unsuitable for the trial;
11. Persistent withdrawal from clinical trials.

### Patient screening

**Fig. 1:**
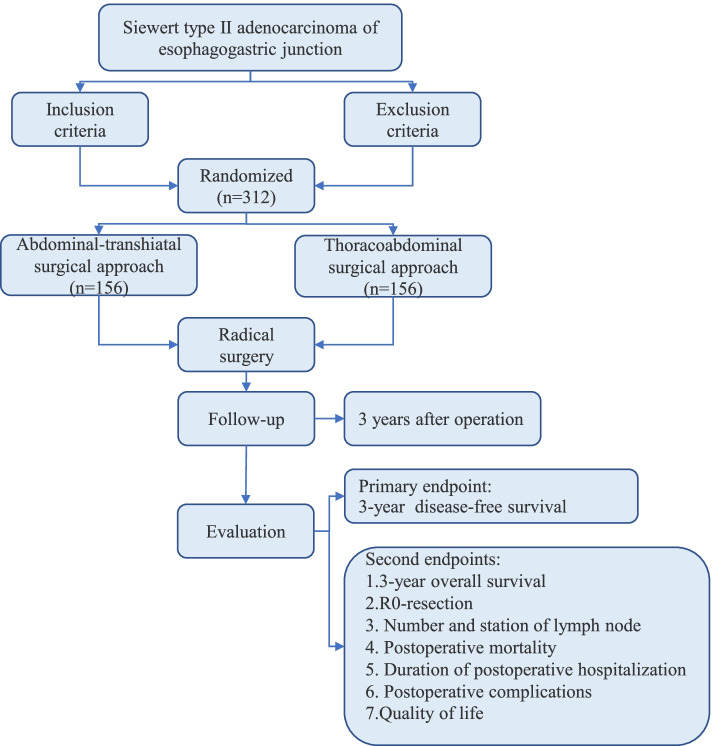
Flow chart

The flow chart of this research is presented in Fig. 1. Multidisciplinary evaluation and standard clinical staging will be done for each patient before enrollment. Based on the anatomical location of the tumors revealed by gastroscopy and the results of the pathological examination of biopsy tissues, the tumors will be classified according to Siewert classification. Imaging examinations (CT, enhanced CT, MRI or PET-CT) will be conducted for determining whether the tumor has distal metastasis and invasion. Complete preoperative preparation also includes anamnesis, demographic information, physical examination, vital signs and laboratory tests.

### Inclusion and randomization of patients

On the completion of the baseline assessment and clinical staging, the criteria for inclusion and exclusion will be validated. Informed consent of all participants must be obtained in this trial. Subsequently, the participants will be further divided into the TH cohort or the RTA cohort in a ratio of 1:1 according to the computer-generated random numbers. Then these participants will receive the operation within 1 week after randomization.

### Follow-up

Follow-up visits among all postoperative patients will be regularly accomplished. The schedule consists of physical examinations, laboratory tests and imaging examinations. Laboratory examinations contain routine blood tests and assessment of tumor biomarkers including CEA, AFP, CA19–9, CA125 and CA153. Imaging examinations (CT, enhanced CT, MRI or PET-CT) will also be performed regularly. Gastroscopy will be done once annually. The follow-up will be performed every 3–6 months for the first 2 years after the operation and every 6–12 months for the following years, according to the schedule (Table 1). Each participant will be tracked for at least 3 years, or until he or she suffers a loss or dies.

**Table 1:** Checklist for collection of necessary clinical data and follow-up schedule of enrolled patients

	Baseline information			Follow-up (months)							
	Preoperation	Operation	Discharge	1	3	6	12	18	24	30	36
Informed consent	**√**										
Demographic information	**√**										
Anamnesis	**√**			**√**	**√**	**√**	**√**	**√**	**√**	**√**	**√**
Oncology history	**√**										
Biopsy	**√**										
Tumor classification	**√**										
Laboratory tests	**√**			**√**	**√**	**√**	**√**	**√**	**√**	**√**	**√**
Physical examination	**√**			**√**	**√**	**√**	**√**	**√**	**√**	**√**	**√**
Inclusion/Exclusion	**√**										
Randomization	**√**										
Postoperative Complication				**√**							
Pathology			**√**								
Chest X-ray/CT	**√**						**√**		**√**		**√**
Gastroscopy	**√**						**√**		**√**		**√**
Abdominal CT/MRI	**√**				**√**	**√**	**√**	**√**	**√**	**√**	**√**
Abdominal ultrasound					**√**	**√**	**√**	**√**	**√**	**√**	**√**
Concomitant Medication	**√**		**√**	**√**	**√**	**√**	**√**	**√**	**√**	**√**	**√**
Surgical information		**√**									

√: Indicates the need to collect the clinical data

This trial began on December 11, 2019. Enrollment will take about 3 years. The follow-up begins when the first patient completes the operation and ends when the last registered patient completes the postoperative 3-year follow-up.

### Data collection and management

When a patient is included in the study, a randomized number will be generated and applied in the database. The patient’s codes are accessible to PI and COI. The study coordinator will collect the clinical data and record it on a unique clinical case report form. Each center assigns a dedicated person responsible for the randomization of the enrolled patients, data entry, proofreading, uploading, etc. The final data is summarized in the central database. Data quality inspectors will regularly inspect and control the quality of data entry and database management.

### Intervention

Depending on randomization results, patients will undergo either the TH or the RTA approach operation. The technical details of both approaches are determined based on the preferences of individual surgeons, on the condition of achieving the key goal of completely tumor resection. Minimally invasive surgery and robotic surgery are allowed if the technology and equipment at the local research center are supported. However, if the tumor cannot be completely removed, the surgeons should promptly change their surgical strategy.

Proximal or total gastrectomy with standard D2 lymphadenectomy will be achieved by the transabdominal approach in the TH group. Distal esophagectomy and mediastinal lymphadenectomy will be done through the transhiatal approach. In the RTA group, the intra-abdominal procedure is the same as in the TH group. In addition, an oblique incision over the right thorax will be made, and esophagectomy and mediastinal lymphadenectomy will be completed through the right transthoracic approach.

Reconstruction methods should be determined by the attending physician based on experience and patients’ individual conditions during the operation. Proximal gastrectomy can be followed by gastric tube construction and esophagogastrostomy. It is recommended that the Roux-en-Y (esophagojejunostomy and jejunojejunostomy) reconstruction will be done following the total gastrectomy.

### Management and quality control

The surgeons participating in this study must meet the following conditions. Each surgeon must have performed the TH and RTA methods in at least 20 cases of AEG surgery and must have passed the qualification examination conducted by three experts in gastrointestinal surgery. Surgeons who want to perform minimally invasive or robotic surgery must prove that they have achieved the operation more than 20 times in a fixed surgical team recently. For a smooth and successful clinical trial, the researchers in all participating institutions must be experts in gastrointestinal surgery to avoid learning curve bias, which is the reason for strict institution invitation and quality control procedures. The pathologist who evaluated the specimens is blind to the surgical approaches because the rate of R0-resection and the status of LNs are the main results of this trial. Patients will be informed of the surgery group they are assigned to. If the patients refuse, they will accept the standard treatment plan of the relevant department.

Throughout the trial, ongoing surgical quality assurance will be undertaken. During each operation, photographs of the surgical details, including the lymphadenectomy region and the fresh specimens following the procedure, will be taken. As a result, these pictures will be used for constant feedback to validate the rationale of the surgical approach, the quality and adequacy of the lymphadenectomy and the integrity of the specimens. They will also be utilized by the research committees to examine and monitor the operation’s quality.

### Perioperative treatment

Nutritional support before surgery will be permitted for patients with nutritional risk followed by the Nutrition Risk Screening 2002 (NRS-2002 > 4). During the perioperative period, antibiotics will be used preventively following relevant national regulations. Postoperative preventive continuous intravenous analgesia will be allowed but is not mandatory. Postoperative fluid rehydration and nutritional support will be implemented based on the experience and rules of the physicians of each participating institution.

### Postoperative adjuvant treatment

Postoperative adjuvant chemotherapy will be principally advised for patients with pathologically confirmed as advanced AEG. The regimen will comply with the Japanese Gastric Cancer Treatment Guidelines 2018 (5th edition) [26].

### Outcome measurements

The primary endpoint of the study is the 3-year DFS. DFS is the timeframe between the operation and recurrence, progression, or death.

The secondary endpoint:

1. 3-year overall survival (OS): OS is the time from operation to death due to any cause or the last follow-up.
2. R0-resection: Patients who undergo radical resection without positive margins;
3. Number and station of lymph nodes (LNs): Detected in postoperative specimens;
4. Postoperative mortality: Death within 30 days after surgery;
5. Duration of postoperative hospitalization: The time from the operation to discharge;
6. Postoperative complications: Postoperative complications include respiratory complications (postoperative pneumonia, pleural effusion, pulmonary insufficiency), cardiac complications (acute myocardial infarction, heart failure, arrhythmia), intra-abdominal bleeding, intra-abdominal abscess, anastomotic structure, anastomotic leakage, chyle leakage, deep venous thrombosis, and wound infection. All postoperative complications will be classified according to the Clavien-Dindo grading system [27];
7. Quality of life (QOL): Using the European Organization for Research and Treatment of Cancer Quality of Life questionnaire (EORTC: QLQ-C30, QLQ-STO22, and QLQ-OG25 after 3, 6, 9, 12, 18, 24, and 36 months).

### Sample size calculation

Following previous studies, the rate of 3-year DFS for AEG patients who underwent surgical treatment is about 41.5% [9]. We assume that there is a significant difference of 0.07 between the TH and the RTA groups. After taking a 10% dropout rate into account, with the conventional 5% type I error (Both sides) and 80% statistical power, the results showed that 156 patients per arm are required. Therefore, 356 patients will be recruited for the study.

### Statistical analysis

IBM SPSS (Version26, Chicago, USA) will be used for statistical analysis. If the continuous variable is normally distributed, it will be presented as the mean (standard deviation, SD) with t-tests, or as the median (interquartile range, IQR) with Mann-Whitney U tests if it is not. Categorical variables will be provided as frequency (N) with percentage (%) and assessed using the chi-square or Fisher’s exact test. The Kaplan–Meier curves with the log-rank test will be used to compare the groups’ survival. A bilateral *p*-value of 0.05 or less is deemed to be statistically significant.

## Discussion

Retrospecting from the past several decades, the incidence of AEG is showing an undoubted upward trend worldwide, and the incidence is also soaring faster than that of other parts of gastric cancer [28, 29]. AEG has a high recurrence and metastasis rate, leading to a poor prognosis [30]. Although the current comprehensive treatment including surgery, chemotherapy and targeted therapy can improve the prognosis to some extent, surgery is the only curable method for AEG [24], Nonetheless, the heated argument of the optimal surgical approach for AEG, particularly ST-II AEG (“true” carcinoma of the cardia) has been ongoing for more than three decades [12]. The treatment for thoracic surgeons usually complies with guidelines of esophagus carcinoma, the TA approach could ensure radical oncology resection for tumor margins and adequate lymphadenectomy, leading to a potential survival advantage [19, 31]. Abdominal surgeons usually consider the TH approach as a minor invasion and quicker recovery method [12, 32].

In the Netherlands, an RCT enrolled patients with Siewert types I and II AEG and compared the efficiency of the right transthoracic and transhiatal approaches. No significant difference was found between the two groups regarding hospital mortality and 5-year survival [33]. Another landmark large-scale RCT in Japanese included Siewert types II and III AEG, which compared the efficiency between the LTA and TH approaches. This trial was closed after interim analysis as the results showed the patients who underwent the LTA approach had more morbidity and no improvement in survival which confirmed in the 10-year follow-up [10, 12]. Recently, a systematic review and meta-analysis by Heger et al. showed that a higher rate of postoperative morbidity was associated with the thoracoabdominal approach for AEG patients compared with the transhiatal approach. However, the most significant limitation of this meta-analysis is the lack of relevant data focusing on ST-II AEG [9]. Recent work by Xing et al. retrospectively collected 211 Siewert type II AEG patients and compared the surgical approaches between the transhiatal and the right thoracoabdominal. As a result, the transhiatal group performed significantly better survival and fewer complications than that in the right thoracoabdominal group, as evidenced by propensity score matching (PSM) analysis [32]. Nevertheless, the credibility of these results should be treated with caution, as for the retrospective design and relatively small size.

To date, there have been no prospective randomized controlled trials regarding surgical therapy for ST-II AEG, leading to ongoing controversial debate for the optimal surgical approach. Our trial (S2AEG) will be the first RCT that focuses on ST-II AEG comparing the safety, feasibility and clinical efficacy between the TH with the RTA approaches. The successful implementation of this study will provide essential data on survival, oncological outcomes and QOL. The purpose of the S2AEG trial is to determine whether the TH or the RTA surgical approach is preferred for the ST-II AEG patients. The TH approach is expected to be equivalent to the RTA approach in terms of survival and oncological outcomes, while still offering an acceptable life quality at the same time. In brief, the results will provide high-level clinical evidence for clinical decision-making regarding the selection of a reasonable surgical approach for ST-II AEG.

## Data Availability

The study’s data and materials will be made accessible upon reasonable request.

## Abbreviations

AEG: Adenocarcinoma of the esophagogastric junction
EGJ: Esophagogastric junction
ST-II AEG: Siewert type II adenocarcinoma of the esophagogastric junction
TH: Abdominal-transhiatal
TA: Thoracoabdominal
RTA: Right thoracoabdominal
LTA: Left thoracoabdominal
QOL: Quality of life
RCT: Randomized controlled trial
LN: Lymph node
DFS: Disease-free survival
CT: Computer tomography
MRI: Magnetic resonance imaging
PET-CT: PET: positron emission computer tomography
ASA: American Society of Anesthesiologists physical status classification
ECOG: Eastern Cooperative Oncology Group
NRS: Nutrition Risk Screening
NYHA: New York Heart Association
FEV1: Forced expiratory volume in the first second
NRS-2002: Nutrition Risk Screening 2002
OS: Overall survival
EORTC: European Organization for Research and Treatment of Cancer
SD: Standard deviation
IQR: Interquartile range
PSM: Propensity score matching
